# Pulmonary nocardiosis in the acquired immunodeficiency syndrome, computed tomographic findings: a case report

**DOI:** 10.1186/1757-1626-2-6642

**Published:** 2009-05-15

**Authors:** Branca Sarcinelli-Luz, Edson Marchiori, Gláucia Zanetti, Claudia Mauro Mano, Flávia Abdalla, Juliana França Carvalho, Carla Assed, Isabella Guedes Santos

**Affiliations:** Department of Radiology, Faculty of Medicine, Fluminense Federal UniversityRua Marquês do Paraná, 303. CEP 24.033.900. Niterói. Rio de JaneiroBrazil

## Abstract

The authors describe a case of pulmonary nocardiosis in a 37-year-old man with acquired immunodeficiency syndrome in treatment with antiretroviral drugs. Clinical symptoms were productive cough, hemoptysis and progressive weight loss. A chest x-ray showed a right upper lobe consolidation while the computed tomography demonstrated consolidation with air bronchogram and airspace nodules. The radiologic findings were unspecific. Consequently, a bronchoscopy with bronchoalveolar lavage was performed, revealing filamentous Gram-positive bacteria (*Nocardia species*). Treatment with trimethoprim-sulfamethoxazole resulted in complete remission of the respiratory symptoms.

## Introduction

Nocardia are aerobic Gram-positive bacteria of the order *Actinomycetales*. They are found in dust, sand, soil and stagnant water. Nocardiosis is mainly an opportunistic infection, but can also affect nonimmunocompromised hosts [1, 2]. Innoculation occurs via inhalation, with over 90% of cases primarily resulting in pulmonary nocardiosis [3]. The most common species causing human infection is the *Nocardia asteroides* complex, which includes *N. asteroides* sensu stricto type VI, *N. farcinica*, *N. nova*, and recently *N. abcessus*. Other human pathogens include *N. brasiliensis*, *N. pseudobrasiliensis*, *N. otitidiscaviarium* (formally *N. caviae*) and *N. Transvalensis* [2].

Pulmonary nocardiosis is an infrequent but severe infection that is most commonly found in immunocompromised patients. Common predisposing factors for nocardial infection include corticosteroid therapy, chemotherapy for neoplasm, and acquired immune deficiency syndrome (AIDS) [4]. Pulmonary nocardiosis is difficult to be diagnosed, and is often mistaken for other lung diseases. We report a case of pulmonary nocardiosis that resembled tuberculosis, in a 37-year old patient with AIDS.

## Case presentation

A 37-year-old Caucasian Brazilian male patient was admitted to the hospital with a 4-month history of cough with purulent sputum, hemoptysis, fever, and weight loss of 5 kg. He was diagnosed with AIDS 2 years earlier, and was taking his antiretroviral medications irregularly. On examination he appeared pale and emaciate, but not cyanotic. His vital signs included a blood pressure of 120/80mmHg, a heart rate of 98 bpm and a respiratory rate of 26 breaths/min. Chest auscultation revealed a condensation syndrome in the upper third of the right hemithorax; cardiac auscultation showed no abnormalities, and the remainder of the physical examination was normal. Laboratory evaluation revealed a red blood cell (RBC) count of 3.46 × 10^6^/mm^3^, hemoglobin level of 10 g/dL, hematocrit of 27%. His WBC count was 11 × 10^3^/mm^3^ (band 4%, mon 64%, eos 1%, lymph 10%). The CD4 + T-cell count was of 228 cell/mL and the plasma viral load was of 12,000 copies/mL.

**Figure 1 A-C:**
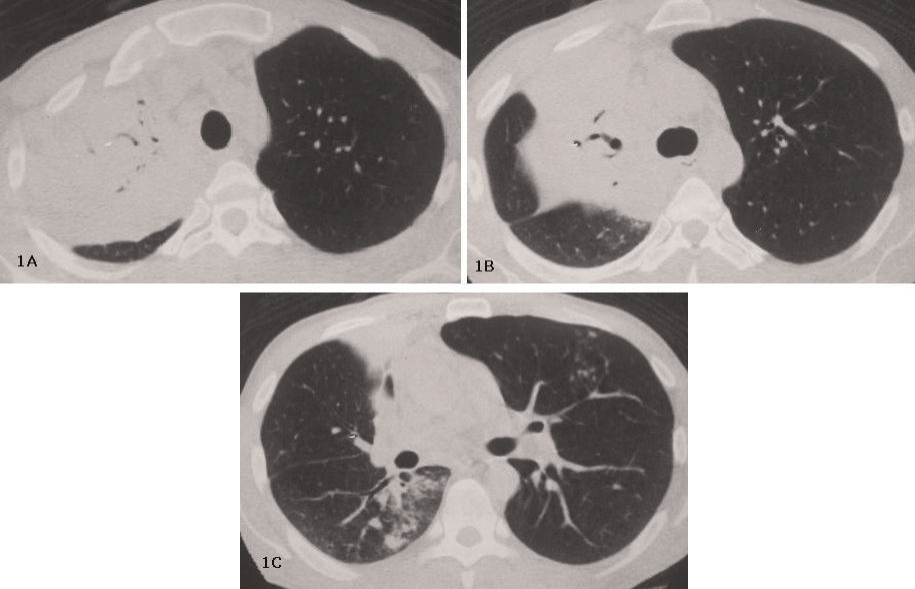
Computed tomography showing extensive area of consolidation with air bronchogram in the right upper lobe. Also note the presence of confluent airspace nodules in the lower part of the consolidation and in the lingular segment **(C)**.

Chest radiograph demonstrated consolidation in the upper lobe of right lung, and computed tomography (CT) revealed the presence of areas of consolidation with air bronchograms and airspace nodules (Figure 1). Several sputum samples were collected and tested for the presence of acid-fast bacilli, but all smears were negative. The patient then underwent bronchoscopy with bronchoalveolar lavage (BAL), and aspirated material was negative for tuberculosis, fungi (including *Pneumocystis jirovecii*), and malignancy. Because of progressive worsening of clinical status, a new bronchoscopy with bronchoalveolar lavage was performed, and this new BAL fluid revealed the presence of filamentous Gram-positive structures compatible with *Nocardia species*.

The patient was started on trimethoprim-sulfamethoxazole, and his antiretroviral therapy was changed to stavudine, lamivudine and nelfinavir. After four weeks of treatment the patient showed a complete regression of symptoms and radiological findings. Six months later the patient was again admitted to the hospital for other complications related to AIDS, and rapidly progressed to death. No autopsy was performed.

## Discussion

Pulmonary nocardiosis is a well-described infection in patients with neoplastic disease, human immunodeficiency virus (HIV) infection, and those receiving treatments with corticosteroids or various chemotherapeutic agents. This disease is a subacute or chronic pneumonia caused by a species of the family *Nocardiaceae*. Seven species have been associated with human disease. *N. asteroides* is responsible for about 70% of infection caused by these organisms [4], and debilitated patients have a 45% mortality rate even with appropriate therapy. Mortality is increased in disseminated disease involving 2 or more organs. There is no age or race predilection. The patients may present with cough, fever, and breathing difficulties [5].

The radiographic appearance of pulmonary nocardiosis is varied and nonspecific. The most commonly described findings include localized consolidation, cavitations, and lobar infiltrative disease with characteristically thick-walled cavities. Computed tomography findings include consolidation with or without cavitation, multiple discrete pulmonary nodules, pleural effusion, and chest wall extension. Notably, AIDS patients diagnosed with pulmonary nocardiosis were found to have more irregular, spiculated nodules, and a higher incidence of cavitary masses [1, 3]. The diverse radiological manifestations of pulmonary nocardiosis reflect its ability to cause both suppurative and granulomatous infection [6].

HIV-related nocardiosis usually appears in patients with advanced immunossupression. Previous studies have reported that between 57% and 68% of patients have AIDS-defining criteria at the time of diagnosis of nocardial infection, and that the CD4+ count is less than 200 cells/μL in 88-100% of these patients [7].

Since the clinical and radiologic manifestations are non-specific, and the microbiological diagnosis is often difficult, it seems likely that, in some patients, pulmonary nocardiosis will be mistaken for other infections, such as tuberculosis or bacterial pneumonia [7].

The diagnosis of nocardiosis often is not considered in a patient with significant pulmonary infection, because the incidence of *Nocardia* is relatively low compared with that of many other organisms. Moreover, *Nocardia* is difficult to culture, and there is no reliable serologic test to detect its presence. Its marked radiographic pleomorphism also tends to exclude it from differential diagnosis of chest film abnormalities, since there are no characteristic findings that bring it to mind [8]. For this reason, nocardiosis should be considered in the differential diagnosis of any chronic pneumonia not responding to the antibiotic treatment [5].

## Abbreviations

AIDS: acquired immune deficiency syndrome
BAL: bronchoalveolar lavage
CT: computed tomography
HIV: human immunodeficiency virus
KS: Kaposi's sarcoma
